# Research progress of biomineralization for the diagnosis and treatment of malignant tumors

**DOI:** 10.3389/fphar.2023.1335019

**Published:** 2023-12-14

**Authors:** Yulu Wu, Xun Pan, Huixu Xie, Lin Que, Xiufa Tang

**Affiliations:** ^1^ West China School of Stomatology, Sichuan University, Chengdu, China; ^2^ State Key Laboratory of Oral Diseases, West China School of Stomatology, Sichuan University, Chengdu, China; ^3^ Department of Oral and Maxillofacial Surgery, West China Hospital of Stomatology, Sichuan University, Chengdu, China

**Keywords:** tumor therapy, CCTC, malignant tumors, diagnostic and treatment, biomometic mineralization, tumor mineralization therapy

## Abstract

Malignant tumors have long been a prominent subject of research in order to foster innovation and advancement in diagnostic and therapeutic modalities. However, the current clinical treatment of malignant tumors faces significant limitations. In light of recent advancements, the World Health Organization (WHO) officially designated malignant tumors as a chronic disease in 2006. Accordingly, maintaining the tumor in a stable state and minimizing its detrimental impact on the body emerges as a potentially advantageous approach to oncological treatment. One emerging strategy that has garnered substantial attention from the academic community is the construction of a biomineralized layer surrounding solid tumors for tumor blockade therapy. This innovative approach is regarded as safe, effective, and long-lasting. This review aims to provide a comprehensive summary of the advancements made in the utilization of biomineralization for the diagnosis and treatment of malignant tumors.

## 1 Introduction

Technological and medical advancements have extended human lifespan, coinciding with increased prevalence of malignant tumors, now a major global mortality cause (Siegel et al., 2023). The exploration and enhancement of diagnostic and treatment approaches for malignant tumors have consistently remained a focal point of research. Among the various manifestations of advanced malignant tumors, bone metastasis stands out as a frequent occurrence, which often induces severe pain, pathological fractures, and nerve damage (Coleman, 2006; Coleman et al., 2020).

Surgical intervention is the principal approach for primary tumors, aiming for complete resection. However, its effectiveness is often limited by tumor spread to adjacent tissues or distant micro-metastases, potentially hindering minimal residual disease progression (Zhao et al., 2016), which may inadvertently impede the natural progression of minimal residual disease (MRD) (Baum, 1996; Baum et al., 1999; Demicheli, 2001; Demicheli et al., 2001). Radiation and chemotherapy are common, yet they carry significant side effects and cancer recurrence risks (Yahyapour et al., 2018).

Current clinical strategies target the primary tumor and aim to strengthen bones to reduce related complications. Pharmacotherapy, especially bisphosphonates, is central in managing bone metastasis. In refractory cases, ablative surgeries with cement filling are used to prevent fractures, improve life quality, and prolong survival. However, both conservative and surgical approaches have limitations, including risks associated with bisphosphonates like inhibited osteogenesis (Cohen et al., 2022) and jaw osteonecrosis (Drake et al., 2008).

On the one hand, the existing treatment modalities face significant challenges in effectively addressing both the primary tumor and multiple metastatic lesions and adds to the complexity of treatment planning. On the other hand, a notable dearth of clinically efficacious treatment approaches persists for managing multiple bone metastases.

Biomedicine research, particularly in biomineralization, has advanced, offering new avenues for malignant tumor treatment. Biomineralization, the process of forming inorganic minerals in organisms, mainly involves nucleation, interface recognition, crystal growth, phase transition, orientation, and nanoparticle assembly. Recently, biomimetic mineralization has become an emerging research field for the design and engineering of organisms with the in-depth study of the biomineralization mechanism and its application scope (Nie et al., 2022).

Recent studies focus on biomimetic mineralization for tumor diagnosis and treatment. Encapsulating tumors in a biomineralization layer has emerged as a novel method for tumor blockade, suppressing growth and metastasis by obstructing tumor vasculature and material exchange (Liu et al., 2022). This method’s sustainability and biocompatibility derive from using organism-derived or imported ions. Theoretically, tumor mineralization therapy has the potential to achieve local *in situ* mineralization of tumors, making it a promising approach for the treatment of primary tumors as well as multiple metastases.

This article reviews biomimetic mineralization advancements in malignant tumor treatment, highlighting unresolved challenges and the need for translating research into clinical applications.

## 2 Biomimetic mineralization for tumor therapy

Over the past few decades, various mechanisms have been postulated to explain the natural processes underlying tumor calcification. Notably, certain studies have indicated that hydroxyapatite nanocrystals are initially synthesized within intracellular vesicles before their subsequent propagation into the extracellular matrix (Arsenau et al., 1991). Others suggest that the calcification process is a direct nucleation by matrix macromolecules on the cell surface (Raggio et al., 1986; Landis et al., 1996; Golub, 2009). Once hydroxyapatite crystals are nucleated, they grow and extend further in the extracellular matrix (Cox and Morgan, 2013). To enable successful tumor mineralization, certain conditions must be satisfied: 1) the presence of ions within the biological tissue or lesion site, 2) the combination of these ions with their corresponding counter-ions, 3) the attainment of a supersaturated concentration of both ions, and 4) the eventual deposition of these ions as a solid phase (Zhou et al., 2019).

In 2016, Zhao et al. introduced the concept and methodology of Cancer Cell Targeting Calcification (CCTC) (Blumenthal et al., 2015), drawing inspiration from the spontaneous biomineralization observed in tumor tissues. This approach combines organic components like folate and inorganic elements such as calcium, creating a hybrid system that disrupts cancer cell functions and induces cell death (Bai et al., 2022). Ectopic calcification leads to the dysfunction and death of tumor cells, resulting in effective tumor suppression, metastasis control, and improved survival outcomes compared to conventional chemotherapy approaches. This method necessitates the supplementary administration of folate and Ca^2+^ at the tumor site. It is important to note that the introduced high concentration of calcium ions, approximately 10 mM, significantly exceeds the physiological range (2.25–2.75 mM). Consequently, safety concerns arise, including the potential for inflammation and hypercalcemia, which may result in severe adverse effects such as cardiac arrest, organ failure, and even fatality (Delektorskaya et al., 2009; Zhao et al., 2016). Therefore, in order to advance the clinical applicability of the clinical application potential of tumor-targeted calcification, Zhao and other researchers recognizing the need for safer and more effective approaches, have undertaken further investigations. Their research focuses on two primary areas: 1) achieving *in situ* enrichment of calcium ions for tumor mineralization at physiological concentrations, and 2) developing calcium ion-targeted release nanoparticles. These endeavors aim to expand the potential of tumor-targeted calcification in clinical settings.

### 2.1 Tumor mineralization at physiological concentrations

**FIGURE 1:**
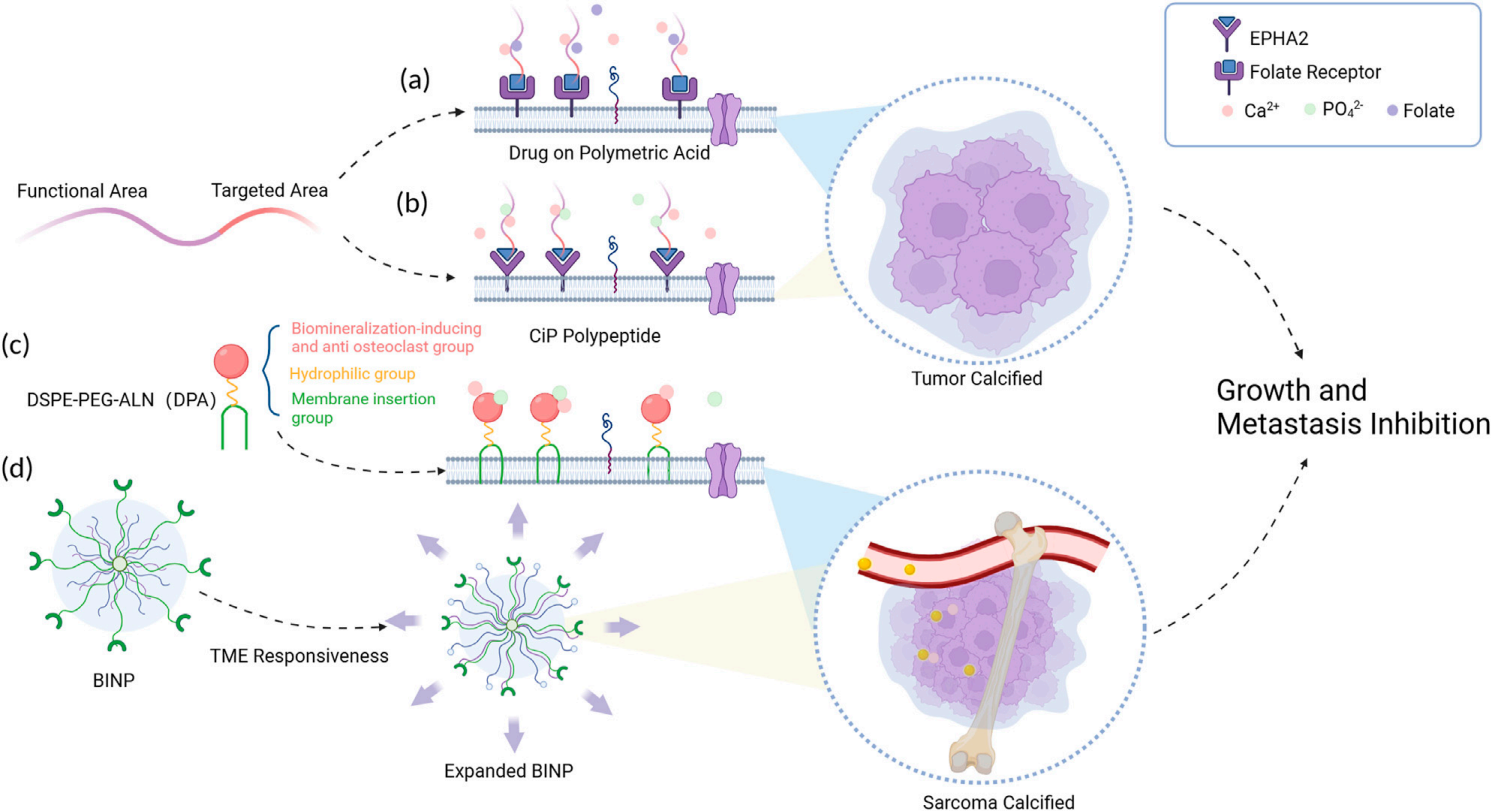
Schematic diagram of tumor mineralization at physiological concentrations. **(A)** Calcium ions and folate molecules were connected to the folate receptors broadly expressed on tumor cells. **(B)** A highly selective calcification-inducing peptide (CiP) was designed to target lung cancer cells. **(C)** The DPA polymer forms a mineral layer on the tumor cell surface with help of its insertion segment. **(D)** BINP responses to the acid tumor micro-environment (TME) and leads to the formation of a biomineralized layer.

In general, the strategy to achieve tumor tissue mineralization at physiological calcium concentrations can be conceptualized as the construction of a functional hybrid system that synergistically integrates organic and inorganic components. The organic constituents of this system primarily comprise structural domains that possess the capability to selectively target tumor cells, thereby enabling enhanced therapeutic selectivity. These domains encompass a diverse range of receptors that are specifically expressed in tumor cells (such as folate receptor (FR), human epidermal growth factor receptor-2 (HER-2), and epidermal growth factor receptor (EGFR) (Delektorskaya et al., 2009)), as well as integrins. Furthermore, the organic components also consider the tumor-specific microenvironment and the vascular niche as important determinants. Additionally, the organic constituents within this hybrid system should encompass structural domains capable of selectively enriching chelated calcium ions from the surrounding tumor microenvironment, thereby facilitating the localized formation of calcium deposits within the tumor tissue. To achieve this, the calcium-inducing units within these structural domains should possess a substantial abundance of strongly negatively charged residues, such as carboxylates, sulfates, and phosphates. By incorporating such elements, the organic components effectively promote the precipitation of calcium ions and contribute to the formation of mineralized structures within the tumor microenvironment (Figure 1).

For instance, in a study conducted by Tang et al., in 2020, a calcification-inducing drug utilizing polysaccharide macromolecules, engineered with folate molecule modifications on polymeric acid, was introduced (Tang et al., 2021). This drug demonstrated the ability to induce tumor calcification under normal physiological concentrations of calcium and phosphate present in the bloodstream. In 2020, Wu et al., 2021 synthesized a highly selective calcification-inducing peptide (CiP) targeting lung cancer cells. The N-terminus of CiP can specifically bind to the membrane of lung cancer cells, and the target paired with CiP is the erythropoietin hepatocellular carcinoma receptor A2 (EPHA2) on the surface of lung cancer cells. The C-terminus contains a calcification-inducing motif that can chelate calcium ions. When administered intravenously, CiP forms a specific precipitation on the surface of lung cancer cell membranes without the need for additional exogenous calcium ion supplementation. In 2022, Liu et al., 2022 s synthesized a functional polymer, DSPE-PEG-ALN (DPA), which consists of three distinct components serving different functions: the cell membrane insertion segment, the hydrophilic segment, and the ion adsorption segment. However, DPA lacks specific tumor cell binding, leading to the development of acid-responsive polypeptide-based biomineralization-inducing nanoparticles (BINP) (Jiang et al., 2022). Upon intravenous administration, the long alkyl chains within BINP undergo assembly within the nanoparticle under neutral conditions, while the tumor microenvironment exhibits weak acidity. In this specific milieu, the histidine structural unit’s imidazole ring in BINP undergoes protonation, transitioning from hydrophobic to hydrophilic, exposing the dodecyl group on the expanded surface of BINP and facilitating its insertion into the tumor cell membrane. Moreover, the bisphosphonate groups present in BINP undergo continuous ion deposition reactions, ultimately leading to the formation of a biomineralized layer on the tumor cell surface.

Tumor-targeted mineralization therapy, at physiological concentrations of calcium ions, induces cancer cell death primarily by disrupting the glycolytic process, influencing cell membrane fluidity, and reducing protein binding capacity (Tang et al., 2021). Notably, cancer cell metabolism is characterized by the ability to extract essential nutrients from a nutrient-limited environment, which is closely associated with the reliance of cancer cells on glycolysis for energy production (Maier and Levine, 2015). Through tumor cell mineralization, the glycolytic process is inhibited, resulting in reduced mitochondrial oxygen consumption and suppression of cancer cell energy metabolism. This disruption of metabolic homeostasis leads to growth arrest and apoptosis in mineralized tumor cells (Arnold et al., 2021). Moreover, mineralization also affects cell membrane fluidity and protein binding capacity. The decreased fluidity of tumor cell membranes leads to membrane rupture and subsequent cell death in cancer cells, significantly restraining their activity and invasive potential (Wu et al., 2021). Additionally, the metastatic capacity and tumorigenicity of tumor cells are markedly diminished (Tang et al., 2021).

### 2.2 Tumor mineralization based on targeted release of calcium ions

Zhang et al. (2019) explored pH-sensitive nanoparticles for tumor mineralization, inducing calcium overload and oxidative stress in cancer cells. In a related study, Ren et al. (2020) developed a novel approach involving the polysaccharide-intervened preparation of hydroxyapatite (HA) hybrid nanoparticles (NPs) with low crystallinity. These HA/ALG NPs are uniquely capable of being specifically up-taken by HeLa cells, facilitating their targeted delivery to the nuclei of tumor cells. Once inside, they release high concentrations of calcium ions locally. This method disrupts calcium signaling and contributes to tumor cell death, highlighting an additional mechanism of tumor mineralization therapy, wherein calcium overload plays a pivotal role in tumor cell death.

Oxidative stress triggers the denaturation and inactivation of calcium channel-related proteins, disrupting calcium channel function and causing the uncontrolled accumulation of Ca^2+^ within cells, effectively inhibiting tumor growth and metastasis *in vivo*. In normal cells, the expression of catalase (CAT) remains unaffected by oxidative stress and serves as a protective mechanism. Conversely, tumor cells exhibit downregulated CAT expression, rendering them more vulnerable to the detrimental effects of excessive intracellular hydrogen peroxide. The subsequent calcium overload irreversibly disrupts calcium signaling, leading to cell death. It has been demonstrated that intracellular calcium overload correlates with the formation of calcium-containing vesicles within cells. Consequently, soluble ions within the cellular environment undergo reduction and precipitation into an amorphous phase, thereby contributing to the calcification of calcium-overloaded tumor cells.

## 3 Biomimetic mineralization for tumor diagnosis

The application of biomimetic mineralization technology in the early diagnosis and prognosis analysis of tumors holds clinical significance.

Firstly, the promotion of mineralized foci formation within tumors facilitates early tumor tissue detection. Currently, the early detection through computed tomography (CT) examinations remains challenging. The CiP synthesized by Wu et al. induces mineralization exclusively on the surface of lung cancer cells, which enhances visual sensitivity in ultrasound and CT imaging, enabling early differentiation between lung cancer and non-tumorous lung nodules. Furthermore, Zhang et al. designed a Prussian blue/calcium peroxide nanocomposite that promotes iron mineralization within tumor cells (Zhang et al., 2021), precipitating Fe(OH)_3_ to facilitate early medical imaging of lung cancer and benign nodules. This approach allows for the detection and prevention of tumor metastasis in the early stages, without the use of toxic drugs, offering a potential solution for the precise management of lung cancer with favorable outcomes. However, it is important to note that the current research in this area predominantly focuses on lung cancer, with limited investigations conducted on other tumor types.

Secondly, spontaneous calcification observed in certain tumors after chemotherapy or radiotherapy has been clinically recognized as a benign prognostic factor in hepatocellular carcinoma (Kim et al., 2020), colorectal cancer (Zhou et al., 2019), lung cancer (Wu et al., 2021) and glioblastoma (Blumenthal et al., 2015). The underlying mechanism is postulated to involve the dysregulation of intracellular calcium signaling caused by oxidative free radicals generated during chemotherapy and radiotherapy, resulting in calcium overload. However, these mechanistic speculations have not yet been experimentally validated.

## 4 Discussion

In summary, this mini review has highlighted recent advancements in animal experiments demonstrating the feasibility of tumor tissue calcification as a promising approach in cancer therapy, particularly in avoiding systemic hypercalcemia (Zhao et al., 2016; Zhang et al., 2019; Tang et al., 2021; Zhang et al., 2021; Liu et al., 2022; Nie et al., 2022). Empirical evidence underscores the benefits of this method, such as disrupting tumor cell growth and metabolism, reducing membrane fluidity and protein binding capacity, inducing tumor necrosis, and diminishing metastatic potential and tumorigenicity (Bai et al., 2022). Additionally, combining tumor tissue calcification with cell-based chemotherapy has shown to maintain high local drug concentrations, enhancing targeted delivery and presenting promising prospects for clinical applications.

Comparatively, tumor calcification therapy offers unique advantages over traditional chemotherapy and radiotherapy, such as targeted treatment and significantly reduced systemic toxicity. In contrast to surgical resection, calcification therapy possesses the distinct capability to manage multifocal or metastatic lesions. Surgical approaches, while effective for excising primary tumors, are typically limited in addressing widespread or disseminated tumor sites. Calcification therapy, conversely, can be employed in these more complex scenarios, providing a non-invasive alternative capable of effectively managing extensive tumor manifestations. Additionally, calcification therapy may potentially be integrated into a sequential treatment paradigm with other therapeutic strategies. This integration could be particularly advantageous in reducing the tumor burden prior to subsequent interventions. By initiating necrosis and diminishing the size of the tumor through calcification, the complexity and invasiveness of later treatments, whether surgical, chemotherapeutic, or radiotherapeutic, could be substantially reduced. This sequential approach not only promises to enhance the overall efficacy of the treatment but also to improve patient outcomes by lessening the trauma and complexity associated with more aggressive therapies.

However, current research in this field has not extensively addressed the comprehensive biocompatibility validation, thus necessitating further studies targeting systemic multi-organ interactions at the theoretical level. In addition, attention must be given to the heterogeneity within different tumor types and even within the same type of tumor. The universality of a single targeting approach across various tumor types requires careful scrutiny. Moreover, challenges such as translating animal model results to human applications and potential side effects need addressing. The transition from laboratory to clinical application involves navigating through clinical trials, regulatory approvals, and ethical considerations, including patient consent for novel treatments. The economic aspects, particularly the cost-effectiveness of tumor calcification therapy, are also vital for its broader adoption.

**FIGURE 2:**
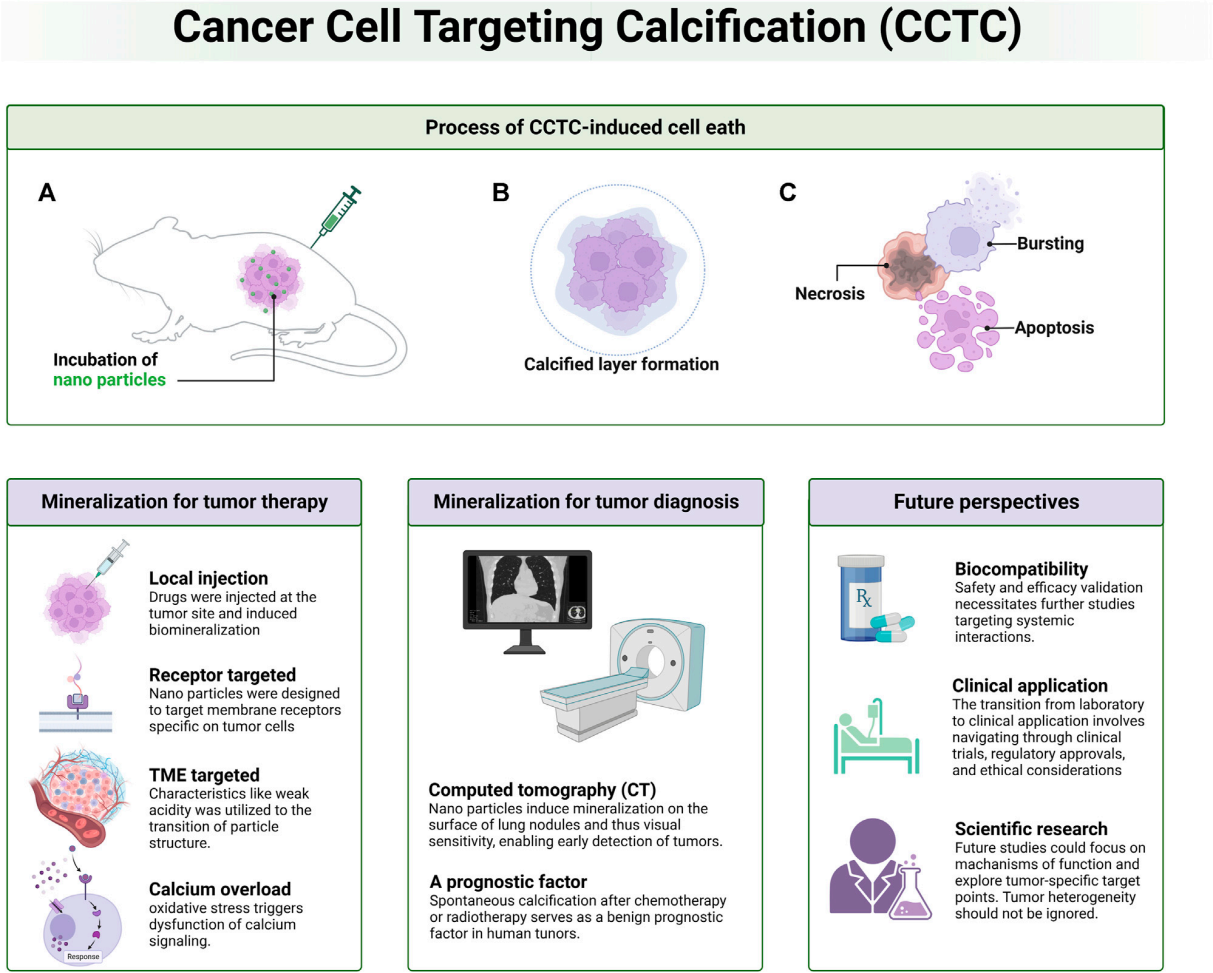
Schematic diagram of overall information related to cancer cell targeting calcification (CCTC). Process of biomimetic calcification therapy, CCTC-based strategies for tumor therapy and diagnosis as well as future perspectives are discussed.

Several key areas in tumor calcification therapy require further exploration (Figure 2). The precise mechanisms by which tumor calcification influences tumor growth, metabolism, and metastasis are not yet fully understood. Gaining insight into these mechanisms is crucial for optimizing treatment strategies. For instance, the effects of partial calcification on the cell cycle and the response of the human immune system to calcified tumor tissue are significant areas for investigation. Additionally, future research trends may involve identifying additional tumor-specific target points and calcification-inducing molecules, thereby expanding the application scope of biomineralization in cancer treatment and diagnosis. Another potential research angle involves tumor stem cells. Since biomineralization therapy does not solely rely on cell surface-specific receptors, it theoretically possesses a robust inhibitory effect on tumor stem cells within the tumor microenvironment. However, this hypothesis requires empirical validation. Interdisciplinary collaborations, such as those between oncologists, bioengineers, and molecular biologists, are essential to propel this field forward.

In conclusion, while tumor tissue calcification presents a novel and promising approach in cancer therapy, its successful translation to clinical practice requires a comprehensive understanding of its mechanisms, careful consideration of its limitations, and a concerted effort across various research disciplines.
